# Upper Limb Deep Vein Thrombosis in Patient with Hemophilia A and Heterozygosity for Prothrombin G20210A: A Case Report and Review of the Literature

**DOI:** 10.1155/2017/7290945

**Published:** 2017-09-25

**Authors:** Fares Darawshy, Yosef Kalish, Issam Hendi, Ayman Abu Rmelieh, Tawfik Khoury

**Affiliations:** ^1^Department of Medicine, Hadassah-Hebrew University Medical Center, Jerusalem, Israel; ^2^Department of Hematology, Hadassah-Hebrew University Medical Center, Jerusalem, Israel

## Abstract

Deep vein thrombosis (DVT) is a rare disease in patients with hemophilia A. We report a case of 22-year-old male with severe hemophilia A who presented to the emergency room with 5-day history of right arm pain that was attributed initially to bleeding event. In the absence of external signs of bleeding or hematoma and normal hemoglobin level, we suspected an underlying DVT. Doppler ultrasonography of the right upper limb revealed thrombosis of the subclavian vein and this was confirmed by CT venography. The d-dimer level was normal and investigations for prothrombotic state revealed heterozygosity for prothrombin G20210A mutation. Treatment with factor VIII and low molecular weight heparin led to successful resolution and marked improvement of his clinical condition.

## 1. Introduction

Hemophilia A is a hereditary hemorrhagic disease characterized by the deficiency of the coagulation FVIII. Due to the bleeding tendencies in hemophilia A patients, the occurrence of spontaneous venous thromboembolism is a rare and even surprising but has been reported in the literature [1–4]. The mechanism of spontaneous venous thromboembolism in hemophilia A patient is unclear due to limited number of cases and there are no clear guidelines for its treatment.

We report a case of 22-year-old male patient with severe hemophilia A who developed DVT in the right upper arm.

## 2. Case Presentation

A 22-year-old male diagnosed with severe hemophilia A on F VIII prophylaxis (recombinant F VIII therapy, 3000 units 3 times a week) self-administered into a peripheral vein of the right arm presented to the emergency room for evaluation of right arm and shoulder pain in the past 5 days. On admission, the patient was hemodynamic and respiratory stable. His physical examination was unremarkable except for right arm tenderness without swelling, erythema, or increased vascular markings.

**Figure 1:**
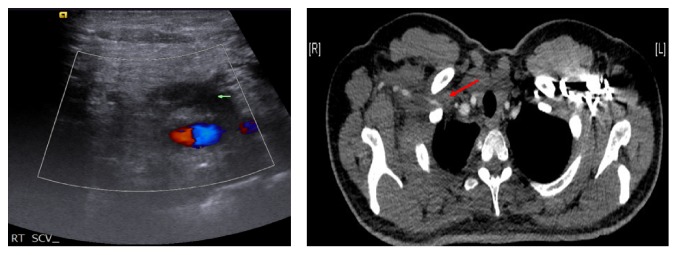
Doppler US and CT scan showing DVT in the right subclavian vein. Arrows refer to deep vein thrombosis.

Prothrombotic workups including protein C activity, protein S activity, antithrombin III level, APC resistance, lupus anticoagulant, anticardiolipin antibodies, factor VIII inhibitor screening, and factor V Leiden defect were all negative, except for heterozygous mutation for prothrombin G20210A; the d-dimer level was normal 0.14 mcg/mL (0–0.5) and coagulation tests revealed low FVIII levels. A Doppler ultrasonography (US) of the right upper limb showed an echogenic thrombosis in the right subclavian vein. CT venography confirmed the diagnosis of right subclavian DVT (Figure 1). All imaging studies ruled out thoracic outlet syndrome.

Further investigations were performed to rule out other causes of DVT; inflammatory markers C reactive protein (CRP) and erythrocyte sedimentation rate (ESR) were within normal range (Table 1). HLA-B51 was performed to rule out Bechet disease and was negative.

The patient was treated with low molecular weight heparin (LMWH) enoxaparin 100 mg once daily (1.5 mg/kg), along with prophylactic FVIII 2000 units per day. This regimen increased FVIII levels up to 49.2% measured 1 hour after FVIII injection and was continued for 2 months.

After two months, the dose of LMWH was reduced to 40 mg and the dose of FVIII was escalated to 3000 units, given three times a week for another 3 months, followed by another six weeks of treatment with 40 mg LMWH and 2000 units of FVIII administered every other day. There was complete resolution of DVT accompanied with significant clinical improvement without recurrence of the thrombosis to date.

## 3. Discussion

Thrombotic events have been rarely reported among patients with hemophilia [1–3]. Goodnough et al. [5] found no thrombotic complications in 178 patients with hemophilia A over a 30-year follow-up period. The exact pathomechanism and the predisposing factors for the occurrence of venous thrombosis in hemophilic patients are still not well established. Kasper found no evidence of thrombosis in major orthopedic operations in patients with hemophilia A [6]. However, Pruthi et al. reported case report of DVT in hemophilia B patient and factor V Leiden following hip fracture surgery [7]. In another recent study, Buckner et al. reported symptomatic venous thromboembolism incidence of 4.3% in hemophilia patients undergoing major orthopedic surgery [8]. Girolami et al. have reviewed all reported patients with hemophilia A and hemophilia B in the literature and found that the administration of factor VIII inhibitor bypassing activity (FEIBA) or recombinant activated FVII (rFVIIa) in patients with inhibitors and surgery was the most frequent risk factor for thromboembolism development in hemophilia A and B patients, respectively, in addition to variable prothrombotic conditions and administration of prothrombin complex concentrate (PCC) [9]. Stewart et al. reported the occurrence of venous thrombosis in a patient with hemophilia A after a long flight [4]. On the other hand, few previous case reports reported venous thrombosis in hemophilia A with no prothrombotic risk factor identified [2, 3]. Our patient developed right subclavian DVT in the arm used for self-injected FVIII prophylactic therapy and was found to have heterozygous mutation for prothrombin G20210A. There were no other identifiable predisposing risk factors as assessed by an extensive investigation that ruled out inflammatory, mechanical obstructive, and neoplastic causes. Prothrombin is a precursor of the serine protease thrombin and is a key enzyme in the process of hemostasis. Prothrombin heterozygosity is related to a single-nucleotide substitution (G to A) at position 20210 in the 3′ untranslated region of the gene encoding prothrombin. Its heterozygous state, 20210A, is a risk factor for the development of deep vein thrombosis [10]. Deep vein thrombosis and cerebral vein thrombosis have been reported in association with heterozygous prothrombin mutation [11, 12]. Interestingly, one may postulate that the low FVIII in our patient of 17% is in favor of bleeding tendency; however, the occurrence of DVT in the setting of low systemic level of FVIII may suggest the strong hypercoagulability potential of heterozygous prothrombin G20210A mutation or, on the other hand, might suggest the hypothesis of FVIII administration as a predisposing factor for local thromboembolism development in hemophilia A patients. Another interesting point is that our patient had normal d-dimer level although he had upper extremity DVT. Normal d-dimer level in two patients with pacemaker and upper extremity DVT was previously reported [13]. In a study for evaluating the usefulness of d-dimer in the evaluation of upper extremity DVT [14], the reported sensitivity was 100% but the 95% confidence interval was 78–100. The specificity was 14%, not surprising given the poor specificity of d-dimer in venous thromboembolism. Based on these previous studies, the use of d-dimer in our case to rule out upper extremity DVT is limited and a Doppler US should be the initial test of choice for upper extremity DVT once suspected.

The treatment of spontaneous venous thrombosis in patients with hemophilia A is not well defined, and the recommended treatment of this condition is based on case reports as different regimens have been used for its treatment. Stewart et al. [4] treated their patient with LMWH for 5 days and tinzaparin for 6 weeks. Dargaud et al. used the unfractionated heparin in addition to FVIII replacement therapy for 1 month [2]. Kashyap et al. used LMWH for 9 weeks [3]. Oral anticoagulation was not used, probably since it increases the risk of bleeding. Bicer et al. [1] treated their patient with LMWH for 2 days followed by warfarin for 6 weeks. No bleeding complications were reported in all cases.

In our case, we treated the patient according to hematological consultation with LMWH on one hand for DVT and with FVIII prophylaxis treating the hemophilia A coagulopathy on the other hand. The regimen was continued for 3 months and then tapered down the administration of LMWH for another six weeks till treatment cessation. This patient was monitored closely and marked clinical and radiological improvement was observed.

In conclusion, spontaneous venous thromboembolism is a very rare and unexpected disease in patients with hemophilia A. Clinicians should be aware about this entity and should consider this differential diagnosis per patient individually especially in the FVIII injected limb. This case shed light on the uncertainty of the mechanism involved in the occurrence of venous thromboembolism in hemophilia A patients especially with low FVIII level. Therefore, further preclinical and clinical trials should be carried out to elucidate the mechanism and treatment of venous thrombosis in patients with hemophilia and to perform larger studies evaluating the exact predictive value of d-dimer in upper limb DVT.

## Figures and Tables

**Table 1:** Laboratory tests results.

Parameter	Result	Normal values
WBC count (*∗*10^9^/L)	8.4	4–10
Platelets count (*∗*10^9^/L)	209	140–400
Hemoglobin (Gr%)	15.1	14–18
PT (%)	73.03	60–100
aPTT (s)	54.7	25–49
INR	1.22	1–1.4
D-DIMER (mcg/mL)	0.14	0–0.5
Factor VIII level (%)	17	70–140
Factor VIII inhibitor screening	Negative	
Protein C activity	Normal	70–120
Protein S activity	Normal	70–140
Antithrombin III level	Normal	
APC resistance	Negative	
Lupus anticoagulant	Absent	
Anticardiolipin antibodies	Absent	
Factor V Leiden defect	Absent	
Prothrombin G20210A	Heterozygote	
ESR	28	1–20
CRP (mg%)	1.42	<0.5
ANA	Absent	
C-ANCA (unit/mL)	8	>18
P-ANCA (unit/mL)	1.6	>18
C3 (mg/dL)	138	90–180
C4 (mg/dL)	27	10–40
